# Supplementary material for: Predicting scale-dependent chromatin polymer properties from systematic coarse-graining
Source: Nat Commun. 2023 Jul 11;14:4108. doi: 10.1038/s41467-023-39907-2 (PMC10336007; doi:10.1038/s41467-023-39907-2)
Supplement: Supplementary file 3 — Reporting Summary [file 41467_2023_39907_MOESM3_ESM.pdf]

nature portfolio

Corresponding author(s): Sangram Kadam and Ranjith Padinhateeri

Last updated by author(s): Apr 6, 2023

Reporting Summary

Nature Portfolio wishes to improve the reproducibility of the work that we publish. This form provides structure for consistency and transparency in reporting. For further information on Nature Portfolio policies, see our Editorial Policies and the Editorial Policy Checklist.

Statistics

For all statistical analyses, confirm that the following items are present in the figure legend, table legend, main text, or Methods section.

n/a	Confirmed	
☐	☒	The exact sample size (n) for each experimental group/condition, given as a discrete number and unit of measurement
☒	☐	A statement on whether measurements were taken from distinct samples or whether the same sample was measured repeatedly
☒	☐	The statistical test(s) used AND whether they are one- or two-sided<br>*Only common tests should be described solely by name; describe more complex techniques in the Methods section.*
☒	☐	A description of all covariates tested
☒	☐	A description of any assumptions or corrections, such as tests of normality and adjustment for multiple comparisons
☐	☒	A full description of the statistical parameters including central tendency (e.g. means) or other basic estimates (e.g. regression coefficient) AND variation (e.g. standard deviation) or associated estimates of uncertainty (e.g. confidence intervals)
☒	☐	For null hypothesis testing, the test statistic (e.g. F, t, r) with confidence intervals, effect sizes, degrees of freedom and P value noted<br>*Give P values as exact values whenever suitable.*
☒	☐	For Bayesian analysis, information on the choice of priors and Markov chain Monte Carlo settings
☒	☐	For hierarchical and complex designs, identification of the appropriate level for tests and full reporting of outcomes
☒	☐	Estimates of effect sizes (e.g. Cohen's d, Pearson's r), indicating how they were calculated

Our web collection on statistics for biologists contains articles on many of the points above.

Software and code

Policy information about availability of computer code

Data collection	LAMMPS (16 March 2018) version was used for chromatin polymer simulations. The codes used in this study are available in the repository: https://github.com/sangramkadam/ chromatin_coarse_graining .
Data analysis	VMD version 1.9.3 was used for the visualization of 3D polymer configurations and the computation of dihedral angles. Gnuplot 5.2 was used for plotting, and Inkscape 0.92 was used for schematic figures. A custom code was used for all other analyses. The codes used in this study are available in the repository: https://github.com/sangramkadam/ chromatin_coarse_graining

For manuscripts utilizing custom algorithms or software that are central to the research but not yet described in published literature, software must be made available to editors and reviewers. We strongly encourage code deposition in a community repository (e.g. GitHub). See the Nature Portfolio guidelines for submitting code & software for further information.

Data

Policy information about availability of data

All manuscripts must include a data availability statement. This statement should provide the following information, where applicable:

- Accession codes, unique identifiers, or web links for publicly available datasets
- A description of any restrictions on data availability
- For clinical datasets or third party data, please ensure that the statement adheres to our policy

Published Micro-C data used in this study is available at the Gene Expression Omnibus (GEO) database with accession number GSE130275. Relevant data generated from this study are included in this article's Figures, text, and supplementary information. Source data are provided with this paper.

Research involving human participants, their data, or biological material

Policy information about studies with human participants or human data. See also policy information about sex, gender (identity/presentation), and sexual orientation and race, ethnicity and racism.

Reporting on sex and gender	Not applicable since there is no research on human subjects in this paper.
Reporting on race, ethnicity, or other socially relevant groupings	Not applicable
Population characteristics	Not applicable
Recruitment	Not applicable
Ethics oversight	Not applicable

Note that full information on the approval of the study protocol must also be provided in the manuscript.

Field-specific reporting

Please select the one below that is the best fit for your research. If you are not sure, read the appropriate sections before making your selection.

☒ Life sciences ☐ Behavioural & social sciences ☐ Ecological, evolutionary & environmental sciences

For a reference copy of the document with all sections, see nature.com/documents/nr-reporting-summary-flat.pdf

Life sciences study design

All studies must disclose on these points even when the disclosure is negative.

Sample size	This is a computer simulation study. The fine-grained model simulation has sampled N=60000 independent polymer configurations. The iterative Boltzmann inversion simulation has sampled N=5000 independent polymer configurations. Model II has sampled N=400,000 independent polymer configurations. The sample sizes were chosen such that the average quantities converged (i.e., average quantities became independent of sample size), and the standard error was very small.
Data exclusions	No data was excluded
Replication	This is a computer simulation study; therefore, replication was checked within the simulation paradigm. The following procedure was used to ensure reproducibility: The total available data was divided into two; both sets produced similar results. Whenever applicable, additional simulations were done to ensure reproducibility.
Randomization	This is a computer simulation study; each chromatin configuration and trajectory is independent. To generate initial configurations, we utilized the random number generator RAN2. The simulation to generate steady-state polymer configurations utilized the random generator in the LAMMPS software.
Blinding	No blinding is performed because this is a computer simulation study where the blinding is not relevant.

Reporting for specific materials, systems and methods

We require information from authors about some types of materials, experimental systems and methods used in many studies. Here, indicate whether each material, system or method listed is relevant to your study. If you are not sure if a list item applies to your research, read the appropriate section before selecting a response.

Materials & experimental systems

n/a	Involved in the study
☒	☐ Antibodies
☒	☐ Eukaryotic cell lines
☒	☐ Palaeontology and archaeology
☒	☐ Animals and other organisms
☒	☐ Clinical data
☒	☐ Dual use research of concern
☒	☐ Plants

Methods

n/a	Involved in the study
☒	☐ ChIP-seq
☒	☐ Flow cytometry
☒	☐ MRI-based neuroimaging
